# Identification of Immune-Related lncRNA Prognostic Signature and Molecular Subtypes for Glioblastoma

**DOI:** 10.3389/fimmu.2021.706936

**Published:** 2021-11-25

**Authors:** Wanli Yu, Yanan Ma, Wenbin Hou, Fang Wang, Wan Cheng, Feng Qiu, Pengfei Wu, Guohua Zhang

**Affiliations:** ^1^ Department of Neurosurgery, Gaoxin Hospital of The First Affiliated Hospital of Nanchang University, Nanchang, China; ^2^ Department of Neurosurgery, The First Affiliated Hospital of Nanchang University, Nanchang, China; ^3^ Laboratory of Medical Genetics, Harbin Medical University, Harbin, China; ^4^ Department of Urology, The Second Affiliated Hospital of Harbin Medical University, Harbin, China; ^5^ Department of Neurosurgery, The Second Affiliated Hospital of Harbin Medical University, Harbin, China; ^6^ The Laboratory of Artificial Intelligence and Bigdata in Ophthalmology, The Affiliated Eye Hospital of Nanjing Medical University, Nanjing, China; ^7^ Oncology Department, Gaoxin Hospital of The First Affiliated Hospital of Nanchang University, Nanchang, China; ^8^ Department of Neurosurgery, The First Affiliated Hospital of USTC, Division of Life Sciences and Medicine, University of Science and Technology of China, Hefei, China; ^9^ Anhui Provincial Stereotactic Neurosurgical Institute, Hefei, China; ^10^ Anhui Province Key Laboratory of Brain Function and Brain Disease, Hefei, China; ^11^ Anhui Provincial Clinical Research Center for Neurosurgical Disease, Hefei, China; ^12^ Central Laboratory, Gaoxin Hospital of The First Affiliated Hospital of Nanchang University, Nanchang, China

**Keywords:** glioblastoma, immune-related lncRNAs, biomarker, prognostic signature, immune infiltration

## Abstract

**Background:**

Glioblastoma multiforme (GBM) is extensively genetically and transcriptionally heterogeneous, which poses challenges for classification and management. Long noncoding RNAs (lncRNAs) play a critical role in the development and progression of GBM, especially in tumor-associated immune processes. Therefore, it is necessary to develop an immune-related lncRNAs (irlncRNAs) signature.

**Methods:**

Univariate and multivariate Cox regression analyses were utilized to construct a prognostic model. GBM-specific CeRNA and PPI network was constructed to predict lncRNAs targets and evaluate the interactions of immune mRNAs translated proteins. GO and KEGG pathway analyses were used to show the biological functions and pathways of CeRNA network-related immunity genes. Consensus Cluster Plus analysis was used for GBM gene clustering. Then, we evaluated GBM subtype-specific prognostic values, clinical characteristics, genes and pathways, immune infiltration access single cell RNA-seq data, and chemotherapeutics efficacy. The hub genes were finally validated.

**Results:**

A total of 17 prognostically related irlncRNAs were screened to build a prognostic model signature based on six key irlncRNAs. Based on GBM-specific CeRNAs and enrichment analysis, *PLAU* was predicted as a target of lncRNA-H19 and mainly enriched in the malignant related pathways. GBM subtype-A displayed the most favorable prognosis, high proportion of genes (*IDH1*, *ATRX*, and *EGFR*) mutation, chemoradiotherapy, and low risk and was characterized by low expression of four high-risk lncRNAs (*H19*, *HOTAIRM1*, *AGAP2-AS1*, and *AC002456.1*) and one mRNA *KRT8*. GSs with poor survival were mainly infiltrated by mesenchymal stem cells (MSCs) and astrocyte, and were more sensitive to gefitinib and roscovitine. Among GSs, three hub genes KRT8, NGFR, and TCEA3, were screened and validated to potentially play feasible oncogenic roles in GBM.

**Conclusion:**

Construction of lncRNAs risk model and identification of GBM subtypes based on 17 irlncRNAs, which suggesting that irlncRNAs had the promising potential for clinical immunotherapy of GBM.

## Introduction

Glioblastoma multiforme (GBM) is the most frequent intracranial primary malignancy in adults. Despite standard treatment, the median survival of GBM patients is less than 14 months (1). In the latest glioma classification, molecular features are considered as classifiers in conjunction with histopathological appearance (2). Emerging biosomics studies have improved the diagnosis and treatment strategies for GBM to some extent but have not yet achieved satisfactory results due to the complex pathogenesis and molecular heterogeneity of GBM. Therefore, more studies are urgently needed to explore the mechanisms involved and to identify novel biomarkers to predict the prognosis and therapeutic effects of GBM.

Long noncoding RNA (lncRNA) is a noncoding RNA with a length of more than 200 nucleotides (3). The discovery of lncRNAs has uncovered new horizons in the pathological processes of multiple diseases, including cancer initiation and progression (4). Recent studies have shown that lncRNAs can influence the tumor immune microenvironment (TIM) by regulating inflammation and participating in immune gene expression (5, 6). For example, lncRNA nuclear-enriched abundant transcript 1 (*NEAT1*) affects cytokine response and induces IGs expression through the regulation of interleukin (IL)-8 transcription (7). LncRNA-*Cox2* participates in inflammatory gene expression in macrophages *via* regulating chromatin complex remodeling (8). Zhao et al. showed that the lncRNA *SNHG14*/*miR-5590-3p*/*ZEB1* positive feedback loop can regulate the PD-1/PD-L1 checkpoint to promote diffuse large B cell lymphoma progression and immune evasion (9). Increasing studies reporting on the mechanism of irlncRNAs in multiple cancers (10), the ambiguous relationship between lncRNAs, and the tumor immune microenvironment have been gradually unveiled. However, the relationship between lncRNAs and tumor immune microenvironment is rarely studied in GBM. Therefore, identification of the irlncRNAs signature may provide a new insight for predicting prognosis and individualized treatment of GBM.

In this study, we identified six key irlncRNA signatures (*H19*, *ST3GAL6-AS1*, *AL162231.2*, *SOX21-AS1*, *AC006213.5*, and *AC002456.1*), which concluded that the risk model indeed had a good predictive outcome. GBM-specific CeRNAs were constructed to predict irlncRNAs targets. GO and KEGG pathway enrichment analysis was used to explore target functions. The PPI network was performed to identify the interactions of proteins translated from mRNAs in the CeRNA network. Furthermore, GS-A showed better prognosis among the identified four GSs (A-D). GSs-specific prognostic value, clinical characteristics, genes and pathways, immune infiltration, and chemotherapeutic drug sensitivity were evaluated. Three hub genes, KRT8, NGFR, and TCEA3, were screened and validated among GSs. These results suggested that the irlncRNAs had the promising potential for clinical immunotherapy of GBM.

## Materials and methods

### Acquisition and Processing of GBM Expression and Clinicopathological Data

RNA-seq transcriptome data of healthy samples were obtained from the GTEx database (11) (http://commonfund.nih.gov/GTEx/). The RNA-seq transcriptome data and clinicopathological data of the GBM samples were downloaded from the TCGA database (http://cancergenome.nih.gov/). Samples and patients with incomplete clinical information were excluded, and conformers are shown in **Table S1**. Two available matrices were merged, normalized with the *limma* package of R software, and obtained the differentially expressed (DE) genes. The input file is FPKM, and the output file is log ^(x + 1)^. The scRNA-seq data of human GBM samples, accession number GSE168004, were obtained from the Gene Expression Omnibus (GEO, http://www.ncbi.nlm.nih.gov/geo/) database. The cutoff criteria were set as | log2 fold change (FC) | > 0.5 and *p* < 0.05.

### Identification of Immune-Related lncRNAs (irlncRNAs)

The immune genes (IGs) list was downloaded from the IMMPORT shared database (12) (https://www.immport.org/) and the Molecular Signatures Database v 7.0 (http://www.gsea-msigdb.org/gsea/index.jsp/). The correlation between genes was calculated to obtain irlncRNAs. Correlation coefficient >0.4 and *p*<0.001 were used as the threshold.

### Establishment of the Immune-Related Risk Prognostic Model

Univariate and multivariate Cox regression analyses were performed to identify significant lncRNAs for construction of the prognostic signature. A risk score was calculated based on each patient’s lncRNAs expression level by the following formula: $Risk\;score\;(RS)=\Sigma_{i=1}^{N}\;Expi*\beta i$ (N is the number of relative lncRNAs, Expi represents the expression value of each lncRNA, and βi is the regression coefficient of the multivariate Cox analysis for the target lncRNA). By setting the median value of the risk score as the cutoff value in the training set and the whole set, GBM patients were divided into high- and low-risk groups. Related files for constructing the immune-related risk prognostic model are displayed in **Table S2**.

### Evaluation and Validation of a Risk Prognostic Model

The predictive ability of the prognostic model was evaluated by a series of analyses: Kaplan-Meier survival analysis, time-dependent ROC curve analyses, univariate Cox regression analysis, and multivariate Cox regression analysis for comparison of the survival between the high- and low-risk groups in the training, testing, and whole cohorts using the R packages *survival* and *survivalROC*. In addition, the signature derived from this study was compared with these three other signature ROC curves (13–15). We analyzed the ROC curve differences between prognostic models and clinicopathological features.

### Construction of a CeRNA Network and a Protein–Protein Interaction (PPI) Network

The miRcode database (16) was performed to match differentially expressed and prognostically related irlncRNAs and miRNAs. Three databases, miRTarBase (17), miRDB (18), and TargetScan (19), were used to predict miRNA target genes. The interactions between miRNAs and lncRNAs or mRNAs were integrated to construct a CeRNA regulatory network. The mRNAs were enrolled in a PPI network through the STRING database (https://string-db.org/) with a confidence score > 0.7. Cytoscape (version 3.8.1) was used to visualize the CeRNA and PPI networks.

### Functional and Pathway Enrichment Analyses

We used the “clusterProfiler” package to perform Gene Ontology (GO) and Kyoto Encyclopedia of Genes and Genomes (KEGG) enrichment analysis of CeRNA network-related IGs to explore potential biological functions and pathways. The cutoff criterion was set at p < 0.05. Additionally, KEGG pathway analysis of KRT8 was performed using the Gene Set Enrichment Analysis (GSEA) software (www.gsea-msigdb.org). The cutoff criterion was set at p < 0.05.

### Identification of GBM Subtypes in Risk Prognostic Model

Unsupervised consensus clustering was conducted to identify a novel immune classification of GBM based on the prognostic irlncRNAs using the *ConsensusClusterPlus* package (50 iterations, resample rate of 80%). The consensus cumulative distribution function (CDF), consensus matrix (CM), and consensus heatmap were performed to determine the optimal number of clusters.

### Analysis of Clinical Characters and Molecular Differences in GBM Subtypes

Survival analysis and valuable clinical information (**Table S3**) were compared between the different subtypes. The Wilcoxon rank test was used to identify differentially expressed molecules among subtypes. The cutoff criteria were set as | log2FC | > 0.3 and *p* < 0.05.

### Immune Microenvironment Exploration for GBM Subtypes Access scRNA-seq Data

The Seurat package was performed for quality control, statistical analysis, and exploration of the scRNA-seq data. The quality control standards were genes detected in >3 cells; cells with >50 total detected genes and cells with ≤5% of mitochondria-expressed genes were included. PCA was used to discriminate available dimensions with a *p* value < 0.05. Then, dimensionality reduction and cluster classification analysis were performed using a t-distributed stochastic neighbor embedding (tSNE) algorithm. The limma package was applied for differential expression analysis to identify the marker genes of each cluster with *p* value < 0.05 and | log2[fold change (FC)] | > 0.5. Based on marker gene populations, different cell clusters were annotated by the singleR package and then manually validated and corrected with the CellMarker database. The corresponding cell surface marker genes for the annotation of cell clusters are listed in **Table S4**.

### Exploration of Candidate Small Molecule Agents

To evaluate the significance of this prognostic model in clinical treatment, the IC_50_ of common administrating chemotherapeutic agents in the GBM dataset TCGA project was calculated. The IC_50_ difference analysis was performed between the high-risk and low-risk groups using the Wilcoxon signed-rank test. Box plots were obtained using *pRRophetic* and *ggplot2* to show the results.

### Preparation for Human GBM Samples

GBM tissues and normal brain tissues were obtained from patients treated at First Affiliated Hospital of Nanchang University who provided informed consent. The study was approved by the hospital’s institutional ethics committee. GBM tissue was collected and immediately stored in an environment at −80°C.

### Quantitative Real-Time RT-PCR (qRT-PCR) Analysis

Total RNA extracted from transfected cells was reverse-transcribed with RT reagent Kit gDNA Eraser (TaKaRa) and detected by SYBR-Green (TaKaRa). The PCR primers are listed in **Table S5**.

### Western Blot Assays

Western blot (WB) assays were performed as described previously (20). The antibodies used are listed in **Table S6**.

### Statistical Analysis

All analysis was carried out by R version 3.6.1 and corresponding packages. Kruskal–Wallis test was used to compare the divergence between multiple groups. Chi-square test or Fisher exact test was used for statistics on clinical information. A Bonferroni test was used to correct the p-value. Kaplan–Meier curves analysis was used to assess survival differences of the subtype. The correlation was determined by Pearson correlation analysis. *p* < 0.05 was regarded as statistically significant.

## Results

### Selection of DElncRNAs, DEimmune genes (DEIGs), and irlncRNAs in GBM

**Figure 1:**
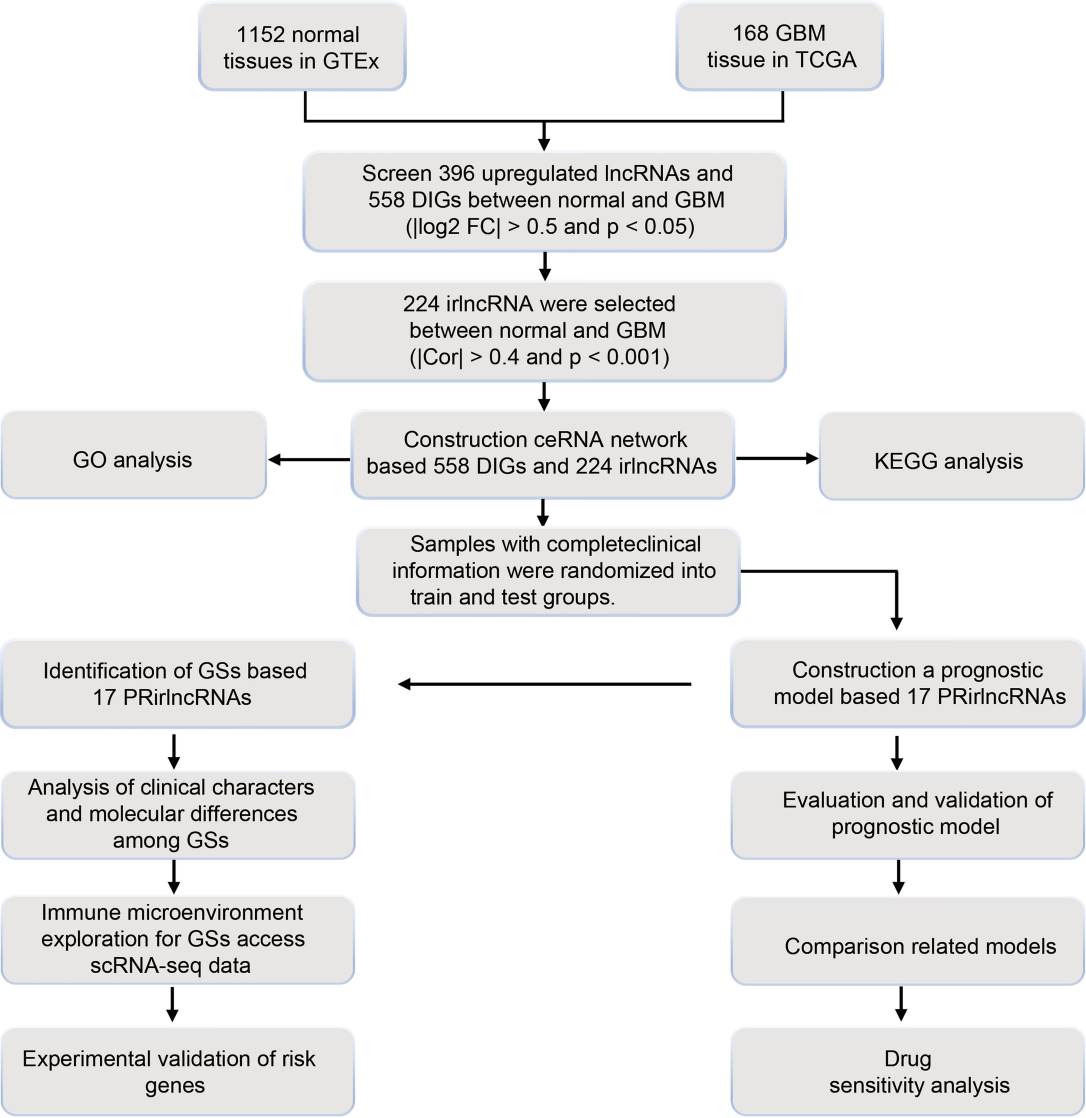
Flow chart of study design.

A filtering flow chart for the study is shown in **Figure 1**. The 1,520 DElncRNAs with 396 upregulated and 1,124 downregulated were identified between normal and GBM tissues. Analogously, we also identified 358 upregulated and 196 downregulated IGs. The corresponding heatmaps are displayed in **Figure S2**. Based on 396 upregulated lncRNAs and 554 DEIGs, 224 irlncRNAs were obtained by correlation analysis (**Tables S7**–**S9**).

### Construction of irlncRNAs Model in GBM

**Figure 2:**
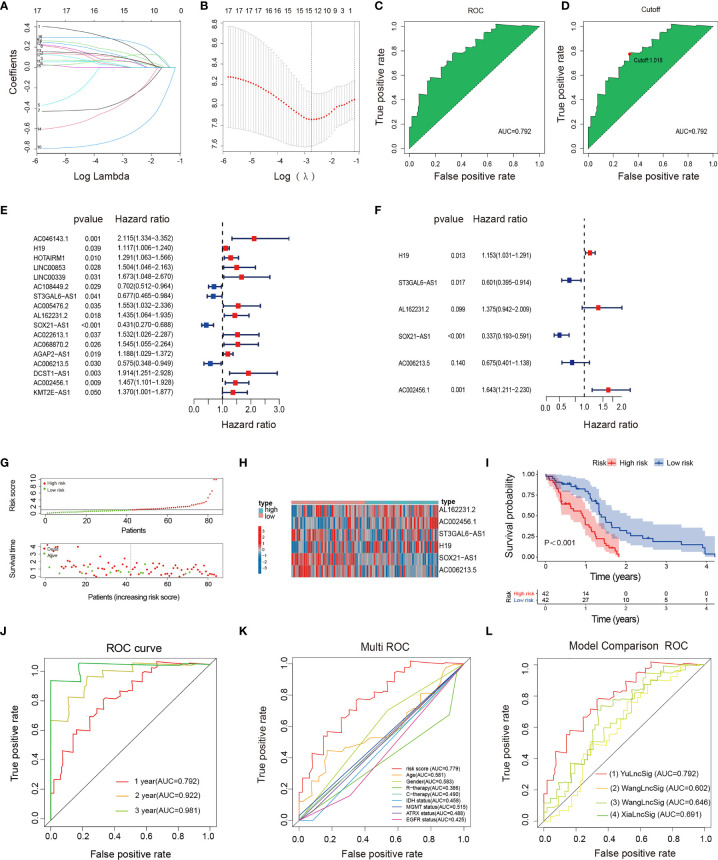
Construction, evaluation, and comparison of a risk signature. **(A)** LASSO coefficient profiles of the 17 irlncRNAs in the training set. **(B)** A coefficient profile plot was generated against the log (lambda) sequence. Selection of the optimal parameter (lambda) in the LASSO model. **(C, D)** The AUC value and cutoff point obtained in the training set. **(E)** Forest plot of 17 irlncRNAs selected by univariate Cox regression analysis associated with GBM survival in the training set. **(F)** Forest plot of six irlncRNAs selected by multivariate Cox regression analysis associated with GBM survival and construction risk model. **(G)** Risk score and survival status analysis of irlncRNAs prognostic signature. **(H)** The expression pattern of irlncRNAs prognostic signature in the low- and high-risk groups. **(I)** Survival analysis of irlncRNAs prognostic signature. **(J)** ROC curve analysis within 1, 2, and 3 years. **(K)** Multivariate ROC curve analysis showing that the superior prognostic performance of the irlncRNAs prognostic model compared to other clinical indicators. **(L)** AUCs of the ROCs for our and the three other gene signatures.

The data of GBM patients were allocated randomly to the training and validation cohort. The 224 irlncRNAs were subjected to univariate Cox regression analysis (**Table S10**) followed by Lasso regression (**Table S11** and **Figures 2A–D**) in the training set to obtain 17 PRirlncRNAs (*p* < 0.05; **Table S12** and **Figure 2E**) and a risk score prognostic model constituted based on 6 key irlncRNAs. The risk score for each sample was calculated based on the expression levels of these six lncRNAs (**Figure 2F**). The coefficient of each gene was calculated by multivariate Cox regression analysis (**Table 1**).


Risk score = (0.14*H19) + (−0.51*ST3GAL6−AS1) + (−0.32*AL162231.2) + (−1.09*SOX21−AS1) + (−0.39*AC006213.5) + (0.50*AC002456.1).

**Table 1:** Coefficients based on a multivariate Cox regression analysis of the selected lncRNAs.

Gene	Coef	HR	HR.95L	HR.95H	p-value
H19	0.143	1.153	1.031	1.291	0.013
ST3GAL6-AS1	-0.509	0.600	0.395	0.914	0.017
AL162231.2	0.319	1.375	0.942	2.009	0.099
SOX21-AS1	-1.087	0.337	0.193	0.591	0.000
AC006213.5	-0.392	0.675	0.401	1.138	0.140
AC002456.1	0.497	1.643	1.211	2.230	0.001

HR, hazard ratio.

### Evaluation and Validation of irlncRNAs Signature in GBM

The irlncRNAs signature is a robust prognostic tool for GBM. Risk curves and scatter plots showed the risk score and survival status of each GBM patient in the training (**Figure 2G**), testing (**Figure S3A**), and total sets (**Figure S1A**). The low-risk group had a lower risk coefficient and mortality than the high-risk group. The heatmap of the irlncRNAs signature in the training (**Figure 2H**; **Table S13**), testing (**Figure S3B**; **Table S14**), and total sets (**Figure S1B**) revealed that GBM with high prognostic scores expressed high-risk irlncRNAs (*H19*, *AL162231.2*, *AC002456.1*), whereas GBM with low prognostic scores expressed protective irlncRNAs (*ST3GAL6-AS1*, *SOX21-AS1*, *AC006213.5*). Based on the median risk score in the training set, GBM patients were divided into high- and low-risk cohorts. Survival curves indicated that patients in the low-risk group had a longer median OS compared with the high-risk group (**Figure 2I**); further examinations were performed in the test (**Figure S3C**) and whole sets (**Figure S1C**) by the same algorithmic cutoff in order to evaluate the accuracy of the prognostic signature. Both groups yielded similar results, suggesting that the prognostic signature was effective. In addition, the promising predictive value for the GBM special model in the training set was demonstrated by ROC curve analysis (**Figure 2J** 1-year AUC = 0.792, 2-year AUC = 0.922, 3-year AUC = 0.981), which validated the results of the model in the testing set (**Figure S3D**; 1-year AUC = 0.703, 2-year AUC = 0.657, 3-year AUC = 0.669) and the whole set (**Figure S1D**; 1-year AUC = 0.744, 2-year AUC = 0.756, 3-year AUC = 0.838). The multi-index ROC analysis revealed that the AUC of the prognostic model was significantly better than those of other clinicopathological indicators (**Figure 2K**) (such as age, gender, therapy, molecular typing, etc.). Compared with three existing lncRNA-related signatures (13–15) (**Figure 2L**), the excellent predictive viability of our model is further demonstrated. Together, these data illustrate the excellent identification of high-risk patients using our model.

### IrlncRNAs Prognostic Model Is an Independent Prognostic Factor for GBM

Univariate and multivariate Cox regression analyses were performed to verify that the irlncRNAs model was an independent prognostic factor for GBM in the training set. The univariate Cox analysis revealed that gender, radiotherapy, MGMT status, and risk score were dramatically associated with the OS (**Figure S4A**), while the multivariate analysis revealed that gender, MGMT status, and risk score were identified as independent prognostic factors (**Figure S4B**).

### Construction of the CeRNA and PPI Network and Functional Enrichment Analysis in GBM

**Figure 3:**
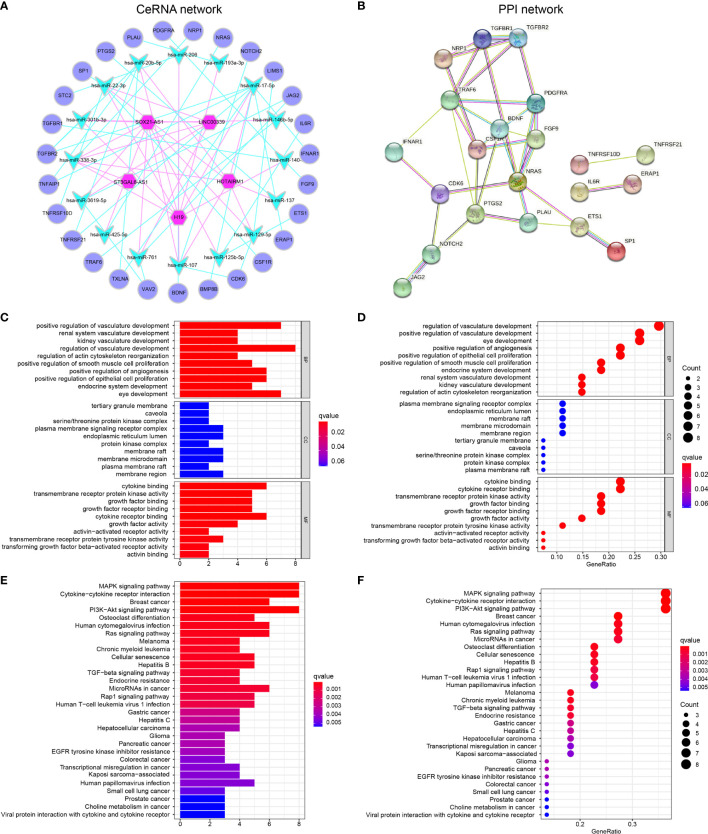
GBM-specific CeRNA network, PPI network, and functional enrichment analysis **(A)** A total of 31 irlncRNAs–miRNA interactions and 35 miRNA–DEIGs interactions construct the lncRNA–miRNA–mRNA regulatory networks. **(B)** PPI network displayed the interactions of proteins translated from IGs in the CeRNA network. **(C, D)** GO enrichment analysis **(E, F)** KEGG pathway enrichment analysis.

Of the 224 irlncRNAs, 17 lncRNAs were associated with prognosis. Based on matching analysis of 17 PRirlncRNAs and 554 DEIGs, a total of 5 irlncRNAs and 16 miRNAs paired into 31 irlncRNAs–miRNA interactions, while 16 miRNAs and 27 DEIGs matched to form 35 miRNA–DEIGs interactions. Finally, 5 irlncRNAs, 16 miRNAs, and 27 DEIGs were used to construct lncRNA–miRNA–mRNA regulatory networks (**Table S15** and **Figure 3A**).

Furthermore, the PPI network was constructed to identify the interactions of proteins translated from mRNAs in the CeRNA network (**Figure 3B**). We found that some genes with high combined score including *TGFBR1-TGFBR2*, *JAG2*-*NOTCH2*, *ETS1-SP1*, *NRAS*-*PDGFRA*, and *BDNF*-*TRAF6* were mainly enriched in the “Human T-cell leukemia virus 1 infection,” “IL-17 signaling pathway,” “TGF-beta signaling pathway,” and “PD-L1 expression and PD-1 checkpoint pathway in cancer pathway.”

GO (**Table S16**) and KEGG (**Table S17**) pathway enrichment analyses demonstrated that the GBM-specific CeRNA network might be involved in the neoplastic process by regulating these biological functions and pathways. GO functional analysis showed that DEIGs involved in the CeRNA network were enriched in BPs, including regulation of vasculature development, response to oxidative stress, and positive regulation of epithelial cell proliferation. The enrichment of MF is mainly related to the membrane signal, and CC is protein binding (**Figures 3C, D**). CeRNA network-related IGs were significantly enriched in KEGG pathways, namely, MAPK signaling pathway, cytokine–cytokine receptor interaction, PI3K-Akt signaling pathway, and multiple cancers (**Figures 3E–F**).

### Four Subtypes of GBM Were Identified and Correlated With Prognosis

**Figure 4:**
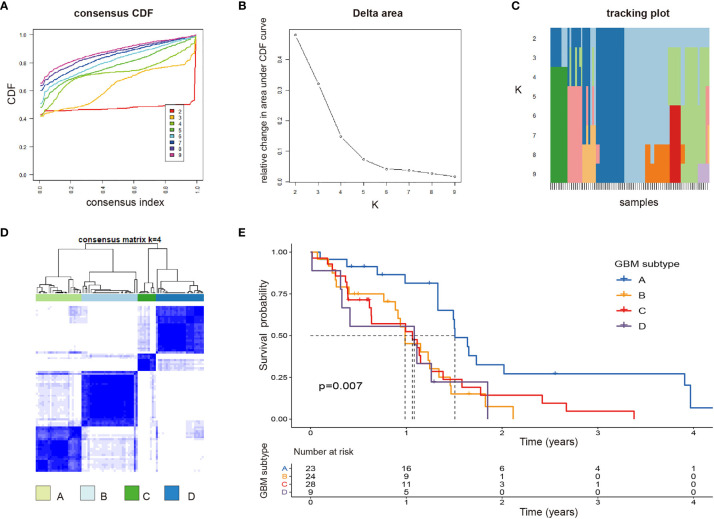
Identification of potential GBM subtypes **(A–C)** Cumulative distribution function curve, delta area, and tracking plot of immune-related lncRNA in GBM. **(D)** Consensus clustering matrix for k = 4, which was the optimal cluster number in the TCGA training cohort. **(E)** Patients in the GBM subtype-A experienced a longer survival time.

Based on 17 PRirlncRNAs, Consensus Cluster Plus was utilized to identify the different subtypes (K = 2-9) among the risk model. According to the cumulative distribution function (CDF) curves, tracking pot, Delta area pot, and CM heatmap (**Figures 4A–D**), when k=4, the sample cluster was stable and robust. As a result, patients could be classified into four GSs (**Table S18**): A (n = 23, 27.4%), B (n = 24, 28.6%), C (n = 28, 33.3%), and D (n = 9, 10.7%). Kaplan-Meier survival analysis indicated that patients with GS-A showed the best OS compared to patients with cluster B, C, or D (*p*=0.007; **Figure 4E**).

### Revelation of Clinical Characters, Molecular Differences, and Pathway Analysis for GBM Subtypes

**Figure 5:**
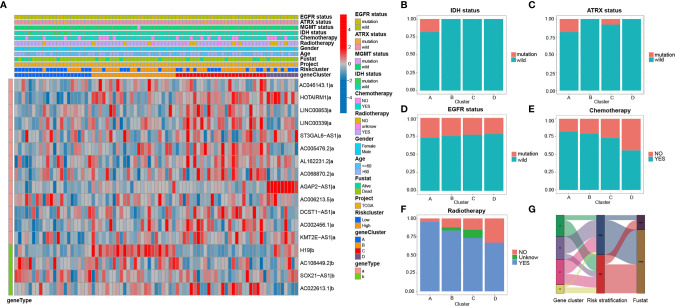
Heatmap and clinicopathological features of four GBM subtypes **(A)** The heatmap and clinicopathological features of the 4 clusters based on the expression patterns of the 17 irlncRNAs in the training set. **(B–F)** Distribution ratio of IDH/ATRX/EGFR status and chemotherapy and radiotherapy in GBM subtypes. **(G)** Sankey diagram showing the prognosis of four GBM subtypes.

Clinicopathological variables and molecular differences were compared among the four subtypes. Heatmap of 17 irlncRNAs illustrated the clinical features and molecular differences among the four subtypes (**Figures 5A**). The results revealed that GS-A patients are characterized by a high mutation rate of genes including *IDH1*, *ATRX*, and *EGFR*, a high rate of chemoradiotherapy, and a high rate of the low-risk group (**Figures 5B–G**).

**Figure 6:**
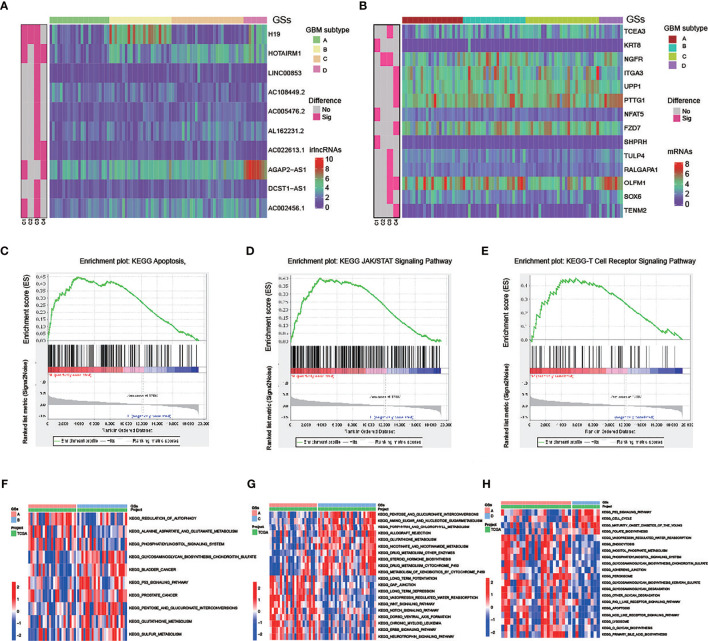
Molecular difference analysis of four GBM subtypes **(A, B)** Heatmaps of 10 differentially expressed irlncRNAs **(A)** and 14 mRNAs **(B)** between the 4 GBM subtypes. **(C–E)** GSEA showing that the functional pathways involved in RKT8 were mainly immune cell- and tumor-related signaling pathways. **(F–H)** GSVA enrichment analysis showing the activation states of biological pathways in GSs.

Subsequently, difference analysis identified 10 lncRNAs (**Table S19**) and 14 mRNAs (**Table S20**) among the four subtypes (**Figures 6A, B**). The results revealed that 6 of the 14 mRNAs were risk genes, and 4 (KRT8, NGFR, TCEA3, and PTTG1) of the risk genes were highly expressed in GBM compared with normal tissues. Thus, these four risk factors, as hub genes, may play an important role in the malignant behavior of GBM.

In addition, patients with GS-A patients are characterized by low expression of four high-risk lncRNA (*H19*, *HOTAIRM1*, *AGAP2-AS1*, *AC002456.1*) and one high-risk gene *KRT8*. GSEA showed that functional pathways involved in RTK8 were mainly immune cell and tumor-related signaling pathways, such as the T cell receptor, apoptosis, or JAK/STATA signaling pathway (**Figures 6C–E**).

GSVA enrichment analysis showed the activation states of biological pathways including the regulation of autophagy, the apoptosis, the Wnt signaling pathway, the NOTCH signaling pathway, the ERBB signaling pathway, the RIG like receptor signaling pathway, and the NOD-like receptor signaling pathway in GS-A (**Figures 6F–H**).

### Exploration of Immune Microenvironment for GBM Subtypes Access scRNA-seq Data

**Figure 7:**
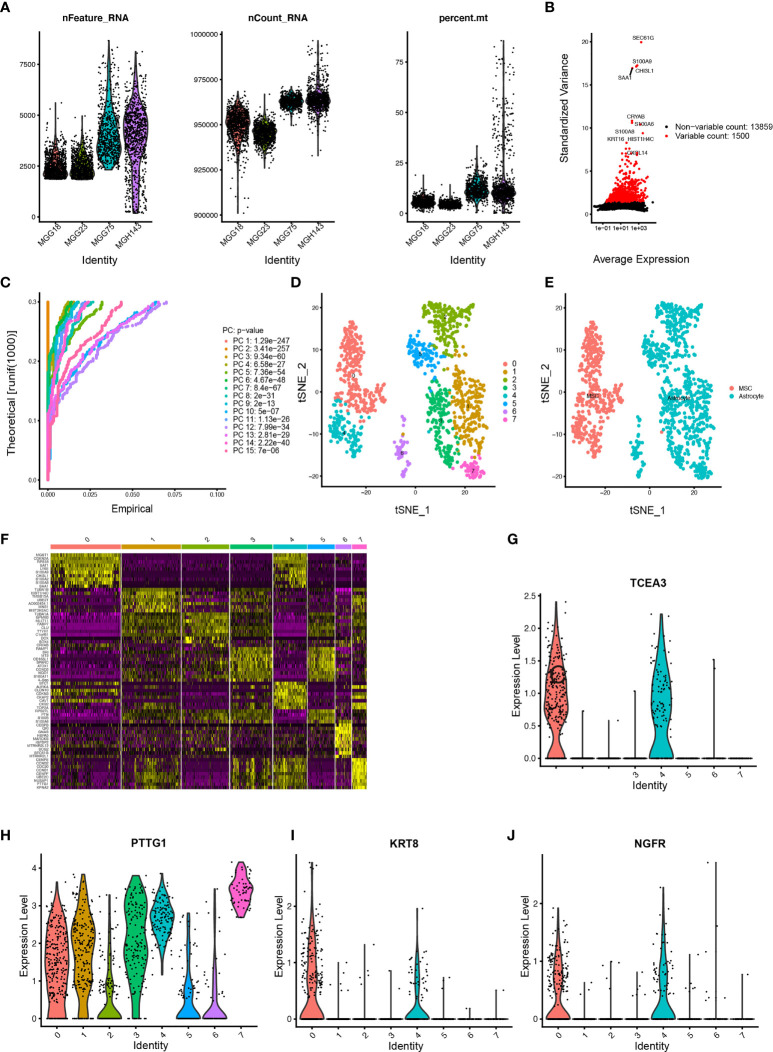
Estimation of tumor-infiltrating cells for GBM subtypes based on scRNA-seq data **(A)** After quality control of the 3,483 cells from the tumor cores of 4 human GBM samples, 1,190 cells were included in the analysis. **(B)** The variance diagram shows 13,859 corresponding genes throughout all cells from GBMs. The red dots represent highly variable genes, and the black dots represent nonvariable genes. The top 10 most variable genes are marked in the plot. **(C)** PCA identified the 15 PCs with an estimated *p* value < 0.05. **(D)** All eight clusters of cells in GBMs were annotated by singleR and CellMarker according to the composition of the marker genes. **(E)** The tSNE algorithm was applied for dimensionality reduction with the 20 PCs, and 8 cell clusters were successfully classified. **(F)** The differential analysis identified 4,210 marker genes. The top 20 marker genes of each cell cluster are displayed in the heatmap. A total of 68 genes are listed beside of the heatmap after omitting the same top marker genes among clusters. The colors from purple to yellow indicate the gene expression levels from low to high. **(G–J)** Expression profiles of the four risk genes in eight cell clusters.

Eight cell clusters with different annotations were identified by scRNA-seq data, revealing cellular heterogeneity in GBM tumors. A total of 4,210 cluster markers were identified from all 8 clusters by differential analysis (**Table S21**). Clusters 0 and 4, containing 398 cells, were annotated as GBM MSCs; clusters 1, 2, 3, 5, 6, and 7, containing 792 cells, were annotated as the astrocytes (**Figures 7A–F** and **Table S22**).

Among the six risk genes of GBM subtypes, two genes, OLFM1 and TENM2, with low expression in GBM were excluded. Searching of the remaining four hub genes (GS-A: KRT8; GS-B: NGFR; GB-C: TCEA3; GB-D: PTTG1) in different GSs with clustering markers revealed that GBM may infiltrate immune cells. KRT8, belonging to Cluster 0, was annotated as GBM MSCs; NGFR, belonging to Clusters 0 and 4, was annotated as GBM MSCs; TCEA3, belonging to Clusters 0, 1, 2, 3, 4, and 5, was annotated as GBM MSCs and astrocyte; PTTG1, belonging to Clusters 2, 3, 4, 5, 6, and 7, was annotated as GBM MSCs and astrocyte (**Figures 7G–J**).

### Screening of the Related Small Candidate Drugs With irlncRNAs Signature

An attempt was made to screen out chemotherapeutic agents that are sensitive to the high-risk group in the TCGA project of the GBM dataset. We found that the high-risk score correlated with a lower half inhibitory concentration (IC_50_) of chemotherapeutic drugs such as Gefitinib (*p*= 0.015) and Roscovitine (*p*= 0.035), whereas it correlated with the higher IC_50_ of axitinib (*p*=0.039) and thapsigargin (*p*=0.0041) (**Figures S4A–D**).

### Validation of the Hub Genes in Clinical Tissues

**Figure 8:**
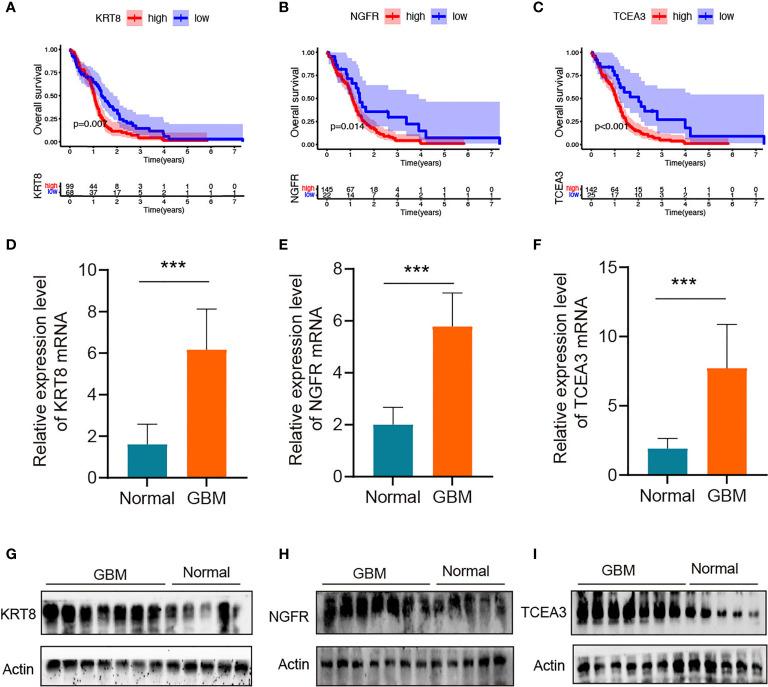
Validation of the hub genes in clinical tissues **(A–C)** Kaplan–Meier survival curves for patients of GBM with high and low gene expression in the TCGA dataset. **(D–F)** qRT-PCR of KRT8, NGFR, and TCEA3 in clinical human GBM samples and normal brain tissues. The expression levels were normalized to β-actin. ***P < 0.001. **(G–I)** The protein expression of KRT8, NGFR, and TCEA3 in clinical human GBM tissues and normal tissues was detected by WB.

The K-M survival curve from the TCGA database was performed to explore the potential role of the individual hub gene in OS. Three of the four hub genes showed significant predictions of poor OS (P < 0.05, **Figures 8A–C**). To further verify the expression level of hub genes in GBM samples, we generated RT-qPCR to calculate the mRNA levels of the three hub genes. As illustrated in **Figures 8D–F**, the expressions of KRT8, NGFR, and TCEA3 were significantly upregulated in GBM tissues compared with normal tissues. Subsequently, WB was used to evaluate the expression level of three proteins. As shown in **Figures 8G–I**, the expression levels of three proteins in GBM tissues were higher than those in normal brain tissues.

## Discussion

GBM cells form a complex tumor microenvironment that supports malignant tumor progression and immune escape (21). Novel immunotherapy within the tumor microenvironment has been uncovered that exerts antitumor immune response *via* targeting immunoregulatory cells or immunosuppressive factors (22). Accumulating evidence suggests that abnormal lncRNAs servers as new markers contribute to antitumor immunoreactivity (23, 24). It is of great significance to understand the tumor immune microenvironment driven by lncRNAs, to construct a clinical prognosis model, and to screen new markers for providing risk stratification and targets for immunotherapy.

In this study, 224 irlncRNAs were analyzed between tumor and normal tissues, 17 PRirlncRNAs were obtained by using the univariate Cox regression analysis, LASSO regression analysis was used to identify 6 key lncRNAs, and multivariate Cox regression analysis was applied to calculate coefficients and construct the risk model. We found that patients in the low-risk group had longer survival than those in the high-risk group. Subsequently, we established forest plots and ROC plots including age, sex, radiotherapy, chemotherapy, gene (*IDH*, *MGMT*, *ATRX*, and *EGFR*) mutation status, and risk scores. By plotting risk heatmap, risk curve, ROC curve, and survival curve, it was concluded that the risk model indeed had a good predictive effect. Meanwhile, we obtained similar results in the validation set.

The immune alterations driven by lncRNAs in GBM have also been preliminarily investigated (20, 25). Among the six key lncRNAs, *H19*, *AL162231.2*, and *AC002456.1* were risk factors for the prognosis of GBM, while *ST3GAL6-AS1*, *SOX21-AS1*, and *AC006213.5* were protective factors. LncRNA *H19* as the first discovered classical regulator lncRNA is involved in the regulation of multiple cancers, including GBM (26, 27). *H19* is overexpressed in glioma tissues, negatively correlates with patient survival, and promotes tumor growth by silencing relevant microRNAs (27, 28). *H19* has a potential reference value for glioma remission and immunotherapy. *ST3GAL6-AS1* and *SOX21-AS1* as protective factors have been reported in cancers, lncRNA *ST3GAL6-AS1* overexpression significantly reduces colorectal cancer cell tumorigenesis and metastasis (29), and lncRNA *SOX21-AS1* significantly suppresses tumorigenesis in cervical cancer (30), oral cancer (31), and GBM (32). Relevant literature reports for *AL162231.2*, *AC002456.1*, and *AC006213.5* are sparse.

Identifying the targets of lncRNAs is a key step in exploring their functions. An immune-related CeRNA network was constructed to predict lncRNAs targets, and a PPI network was used to evaluate the interactions of translated proteins from mRNAs in the CeRNA network. The CeRNA network enabled not only a deeper understanding of the communication between RNAs and a more comprehensive analysis of the complex gene interactions underlying carcinogenesis but also the identification of novel biomarkers. Among the prognostic biomarkers involved in the GBM-specific CeRNA and PPI network, the most significant difference gene was *PLAU *(*e*ncoding urokinase-type plasminogen activator; uPA), which was overexpressed, was the target of lncRNA *H19*, and was enriched in the KEGG pathway, namely, MicroRNAs in cancer, NF-kappa B signaling pathway, transcriptional misregulation in cancer, and proteoglycans in cancer pathways. Moreover, the protein pair with the highest combined score was *TGFBR1–TGFBR2*. *PLAU* is frequently upregulated in GBM (33, 34) and promotes cell invasion by *PLAUR* (*PLAU* receptor) binding and activation of extracellular proteases (35). *TGFBR1* and *TGFBR2* have been identified in GBM as a *TGF*‐*β* signaling upstream receptor (36), which has been well known to be a key regulator of migration phenotype in GBM cells (37). In our analysis, lncRNA *H19* may exert biological activity by targeting miR-193a-3p to regulate gene *PLAU* expression. Therefore, it is meaningful to construct immune-related CeRNA and PPI networks in GBM to mine novel biomarkers, predict prognosis, and guide therapy.

In addition to identifying candidate biomarkers in GBM, GSs are also the key to improve personalized treatment (38). Based on 17 PRirlncRNAs, we can classify GBM patients into 4 GSs (A-D). Then, we assessed subtype-specific prognostic values, clinical characteristics, genes and pathways, immune infiltration, and chemotherapeutic drug sensitivity. Our results revealed that GS-A patients displayed the most favorable prognosis, which were characterized by a high mutation rate of genes including *IDH1*, *ATRX* and *EGFR*. Previously published reports indicated that *IDH*, *ATRX* and *EGFR* mutation status significantly influenced the prognosis of glioma patients (39). Such as, *IDH* mutations are frequent in infiltrating astrocytomas (grades II and III) and secondary *GBMs* (1). Primary GBMs typically lack *IDH* mutations and demonstrate *EGFR*, *PDGFRA*, *TP53*, *PTEN*, *NF1*, and *TERT* promoter mutations (40). These classical biomarkers have been integrated into multiple classification schemes and applied to an accurate clinical decision-making process. We observed that GBM with GS-A were characterized by four high-risk lncRNAs (*H19*, *HOTAIRM1*, *AGAP2-AS1*, and *AC002456.1*) and one high-risk mRNA *KRT8* with a low expression level. Among these lncRNAs and mRNAs, lncRNA *HOXA* transcript antisense RNA myeloid-specific 1 (*HOTAIRM1*) participates in the reprogramming of chromatin organization and proliferation and metastasis of cancer (32), which has been found to be highly expressed in a variety of tumors including GBM (41). LncRNA *AGAP2* antisense RNA 1 (*AGAP2-AS1*), transcribed from a gene located at 12q14.1, a novel cancer-related lncRNA, was dysregulated in cancers (42). In GBM, lncRNA *HOTAIRM1* (43, 44) and *AGAP2-AS1* (45, 46), as oncogenic factors, promoted tumorigenesis, predicted a poor clinical outcome, and were potential biomarkers and therapeutic targets. Keratin 8 (KRT8), a major component of the intermediate filament cytoskeleton, promotes tumor progression and metastasis of various cancers (47–49). In our analysis, GS-A was positively correlated with autophagy, the apoptosis, the Wnt signaling pathway, the NOTCH signaling pathway, the ERBB signaling pathway, the RIG like receptor signaling pathway, and the NOD-like receptor signaling pathway. KRT8 may exert its biological activity through regulating the T cell receptor, apoptosis, or the JAK/STATA signaling pathway.

The efficacy of immunotherapy strongly depends on intertumoral tumor-infiltrating immune cells (50). Combining the risk gene of GSs and scRNA-seq data reveals poor prognosis GBM tumor-infiltrating immunoreactive cells. Park et al. demonstrated that high expression on macrophage signatures of GBM patients predicted suboptimal survival (51), which was consistent with our analysis. We observed that the major tumor-infiltrating immune cells in GBM with poor prognosis were MSCs and astrocyte. Radfar et al. demonstrated that nonspecific activation of CD4^+^ T cells dramatically enhanced the cytotoxicity of four chemotherapeutic agents including TMZ, paclitaxel (Pax), Carbo, and 5-FU in cancers (52). Patients with more infiltrated CD8^+^ T cells had a better response to pembrolizumab treatment than those with less infiltrated cells (53). Our model suggested that high risk was associated with sensitivity to chemotherapeutics such as gefitinib and roscovitine, and GS-A patients in low risk were more sensitive to axitinib and thapsigargin.

Our survival analysis and *in vitro* study showed that three of the four hub genes showed significant predictions of poor OS, and the mRNA and protein levels of KRT8, NGFR, and TCEA3 were significantly upregulated in GBM tissues compared with normal tissues. The above results indicated that these proteins encoded by the hub genes may play a feasible oncogenic role in GBM.

## Conclusion

In this study, based on 17 PRirlncRNAs, we not only constructed a six-key irlncRNAs prognostic signature but also identified four subtypes of GBM, which had a potential prognostic value. In GBM, lncRNA *H19* may exert biological activity by targeting miR-193a-3p to regulate gene *PLAU* expression; KRT8, NGFR, and TCEA3 may stimulate novel strategies for immunotherapy of GBM patients. Interestingly, KRT8 may exert its biological activity through regulating the T cell receptor, apoptosis, or the JAK/STATA signaling pathway.

### Highlights

1. Transcriptome and clinical information from 168 GBM samples was employed to screen 17 immune related lncRNAs (irlncRNAs) associated with prognosis.
2. 17 PRirlncRNAs were screen to construct a signature of 6 key irlncRNAs, which showing a good predictive effect, and similar results in the validation set.
3. GBM-specific immune CeRNA and PPI networks were constructed to predict lncRNAs targets and evaluate the interactions and functions of immune mRNAs translated proteins based on 17 PRirlncRNAs.
4. Four GBM subtypes (A–D) were identified based on 17 PRirlncRNAs, and we evaluated subtype-specific prognostic values, clinical characteristics, genes and pathways, immune infiltration, and chemotherapeutics efficacy.
5. Construction of the lncRNAs risk model and identification of GBM subtypes under immune environment, suggesting that KRT8, NGFR, TCEA3, and irlncRNAs had promising potential for clinical immunotherapy of GBM.

## Data Availability Statement

The datasets presented in this study can be found in online repositories. The names of the repository/repositories and accession number(s) can be found in the article/**Supplementary Material**.

## Ethics Statement

Consent for participation for all patients was obtained through The Genotype-Tissue Expression (GTEx) Project, The Cancer Genome Atlas (TCGA) Project, and the Gene Expression Omnibus (GEO) database. Paraffin-embedded GBM tissues and normal brain tissues were obtained from patients who provided informed consent under an Institutional Ethics Committee-approved study from the First Affiliated Hospital of Nanchang University.

## Author Contributions

All authors contributed to the analysis of data in this study. Conception and design: GZ, FQ and PW. Acquisition, analysis and interpretation of data: WY, WH and FW; Writing, review, and/or revision of the manuscript: WY; Administrative, technical, or material support: YM and PW. Study supervision: YM and WC. All authors contributed to the article and approved the submitted version.

## Funding

This work was supported by the Project of Nanchang Science and Technology Support Plan of Jiangxi Province, China (HONGKO Zi [2021] 129) and the National Institute of Health Grant (5R01AR067319-04. 09/2015-07/2020).

## Conflict of Interest

The authors declare that the research was conducted in the absence of any commercial or financial relationships that could be construed as a potential conflict of interest.

## Publisher’s Note

All claims expressed in this article are solely those of the authors and do not necessarily represent those of their affiliated organizations, or those of the publisher, the editors and the reviewers. Any product that may be evaluated in this article, or claim that may be made by its manufacturer, is not guaranteed or endorsed by the publisher.

## Abbreviations

GBM, glioblastoma multiforme; lncRNAs, long noncoding RNAs; irlncRNAs, immune-related lncRNAs; GSs, GBM subtypes; PRirlncRNAs, prognostically related irlncRNAs; GTEx, genotype-tissue expression; TCGA, The Cancer Genome Atlas; CGGA, Chinese Glioma Genome Atlas; GEO, Gene Expression Omnibus; OS, overall survival; IGs, immunity genes; NEAT1, nuclear-enriched abundant transcript 1; TIM, tumor immune microenvironment; DE, differentially expressed; IG, immune genes; PPI, protein–protein interaction; GO, Gene Ontology; KEGG, Kyoto Encyclopedia of Genes and Genomes; GSEA, Gene Set Enrichment Analysis; ROC, receiver operating characteristic; TMA, temozolomide; FC, fold change; CDF, cumulative distribution function; CM, consensus matrix; BP, biological processes; MF, molecular function; CC, cellular component; IDH, isocitrate dehydrogenase; MGMT, O6-methylguanine DNA methyltransferase; EGFR, epidermal growth factor receptor; HOTAIRM1, HOXA transcript antisense RNA myeloid-specific 1; AGAP2-AS1, AGAP2 antisense RNA 1; KRT8, keratin 8; NGFR, nerve-growth-factor receptor; TCEA3, transcription elongation factor A3; PTTG1, pituitary tumor transforming gene 1.
