# Supplementary material for: Identification of Immune-Related lncRNA Prognostic Signature and Molecular Subtypes for Glioblastoma
Source: Front Immunol. 2021 Nov 25;12:706936. doi: 10.3389/fimmu.2021.706936 (PMC8657607; doi:10.3389/fimmu.2021.706936)
Supplement: Supplementary file 1 [file DataSheet_1.docx]

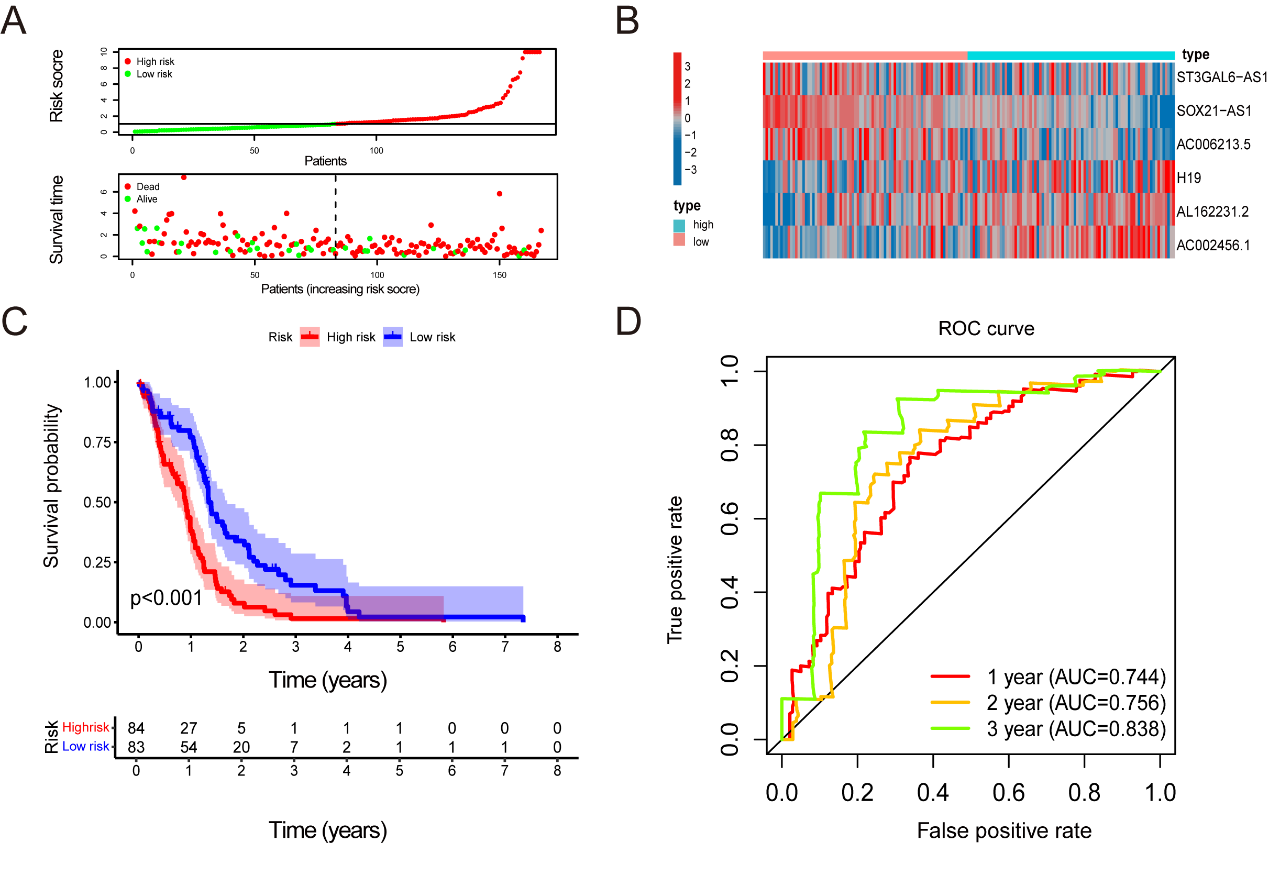


**Figure S1** | Validation of lncRNAS prognostic model in TCGA whole set (n=168);

(**A**) Survival status and risk score analysis of DMDGs prognostic signature;

(**B**) The expression pattern of risk prognostic signature in low and high-risk groups;

(**C**) Survival analysis of risk prognostic signature;

(**D**) The ROC curve analysis within 1-, 2-, and 3-year.


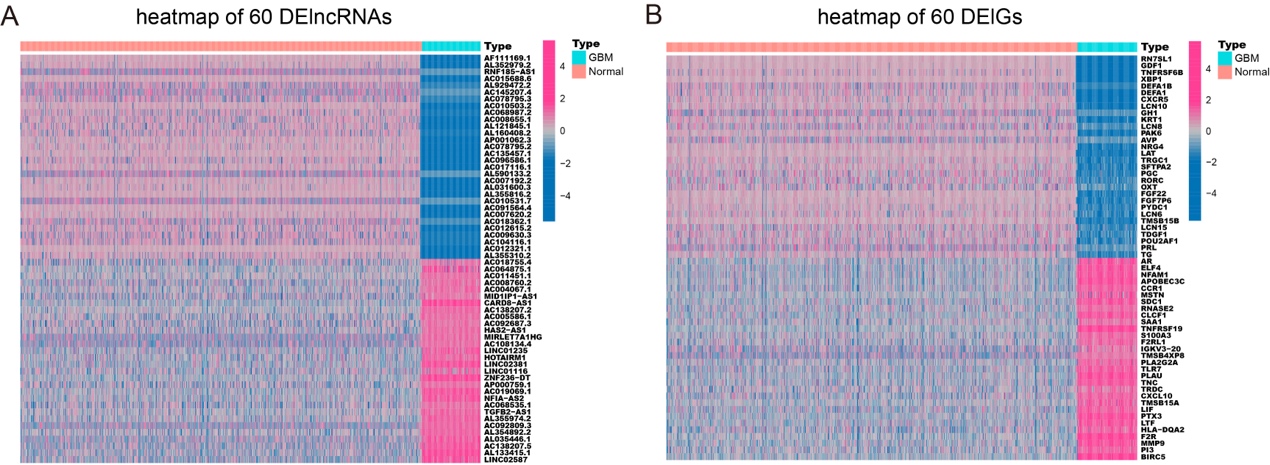


**Figure S2 | heatmap of 60 DElncRNAs and DEIGs**

(A) Heatmap exhibits the expression levels of the DElncRNAs.

(B) Heatmap exhibits the expression levels of the DEIGs. Colors from pink to blue denote the trend from upregulated to downregulated genes between normal and GBM tissues.


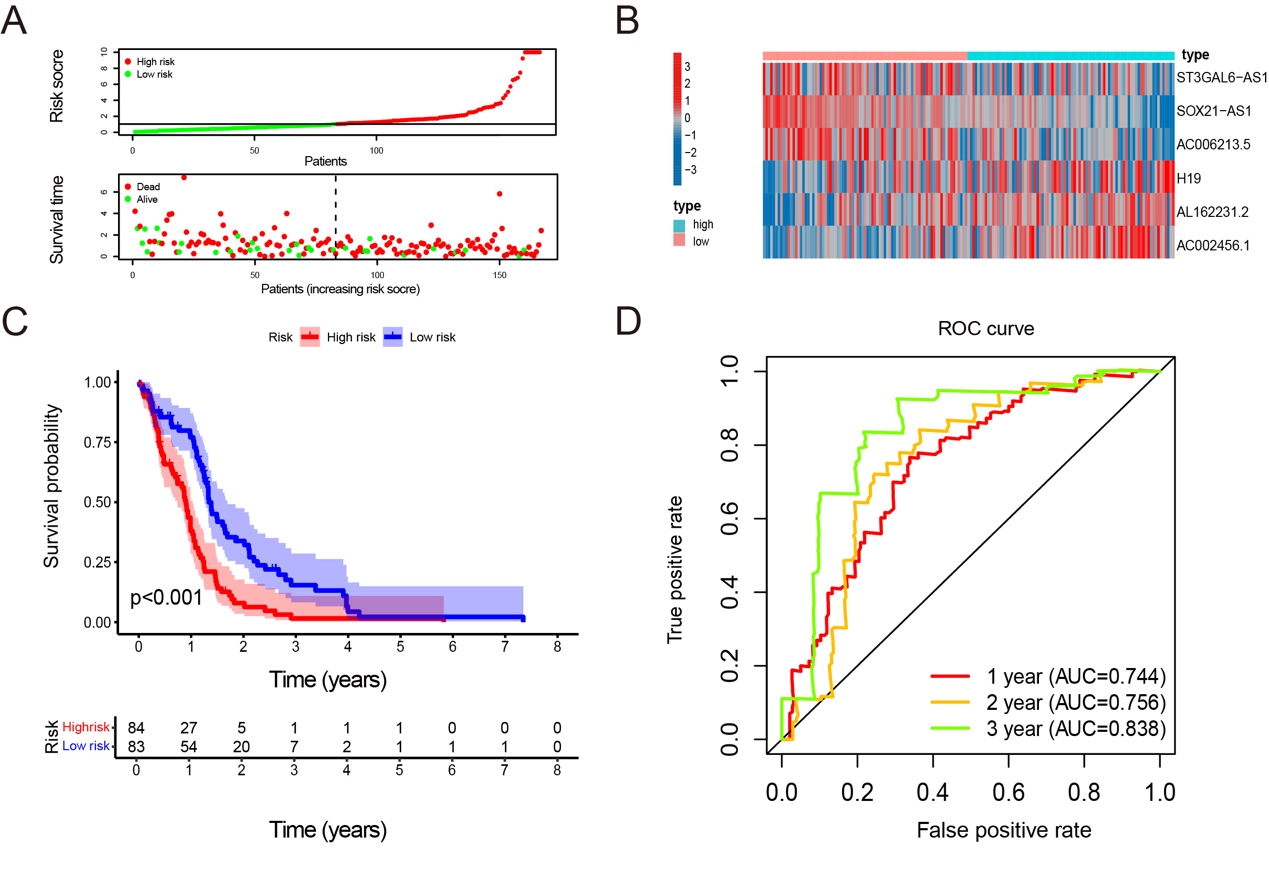


**Figure S3** | **Validation of the risk score model in TCGA testing cohort**

(**A-C**) Risk score analysis, survival status analysis, Heatmap of the 6-gene expression pattern, and survival analysis in low- and high-risk groups in TCGA testing set.

(**D**) ROC curve analysis in TCGA testing set.


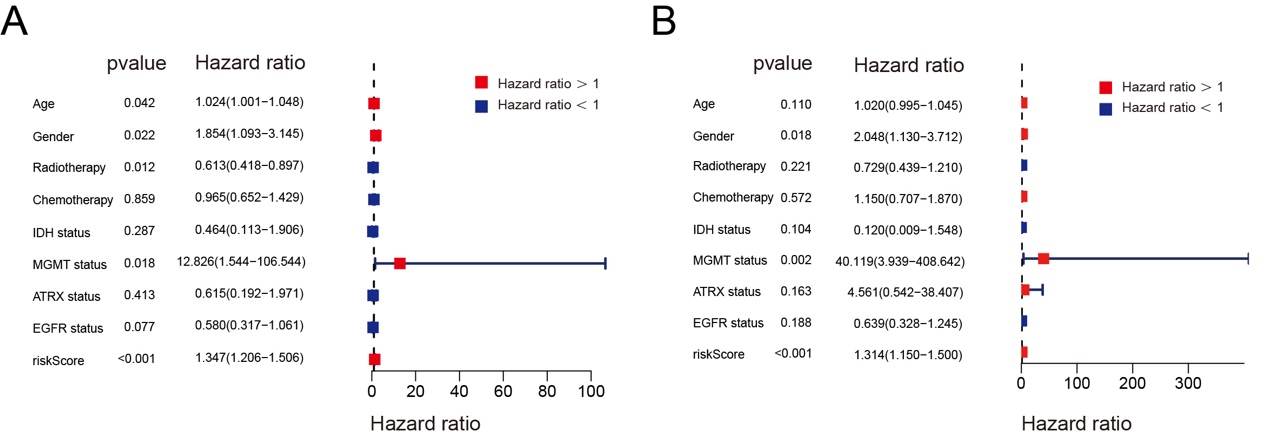


**Figure S4 | IrlncRNAs prognostic model is an independent prognostic factor**

(**A**) Univariate analysis of the risk score and other clinical information.

(**B**) Multivariate analysis of the risk score and other clinical information.

**
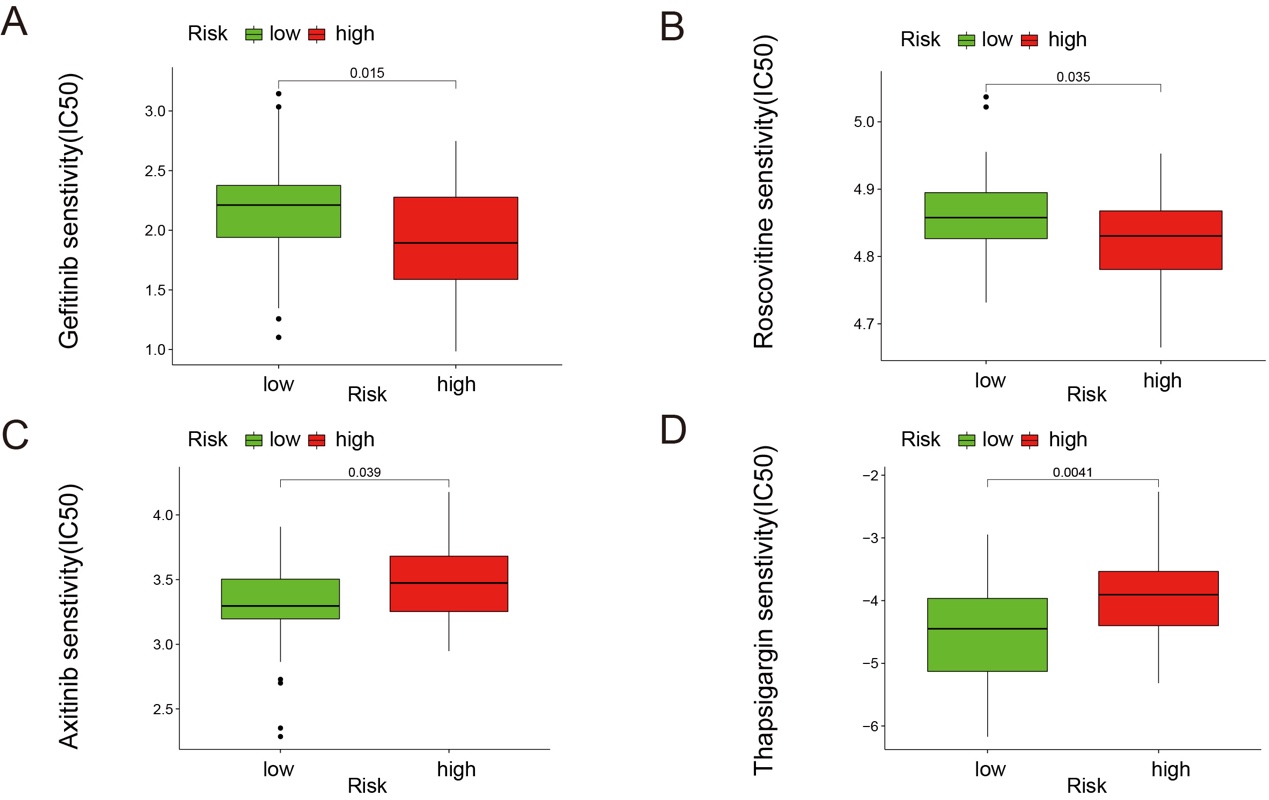
**

**Figure S5 | drug sensitivity analysis**

(**A-D**) The model acted as a potential predictor for chemosensitivity as low risk scores were related to a higher IC_50_ for chemotherapeutics such as Gefitinib and Roscovitine, whereas they were related to a lower IC_50_ for Axitinib and Thapsigargin.
